# Health inequalities in hypertension and diabetes management among the poor in urban areas: a population survey analysis in south Korea

**DOI:** 10.1186/s12889-016-3169-8

**Published:** 2016-06-10

**Authors:** Young-Jee Jeon, Chung Reen Kim, Joo-Sung Park, Kyung-Hyun Choi, Myoung Joo Kang, Seung Guk Park, Young-Jin Park

**Affiliations:** Department of Family Medicine, Ulsan University Hospital, University of Ulsan College of Medicine, Ulsan, Republic of Korea; Department of Rehabilitation Medicine, Ulsan University Hospital, University of Ulsan College of Medicine, 877, Bangeojinsunhwando-ro, Dong-gu, Ulsan, 44033 Republic of Korea; Department of Family Medicine, Dong-A University College of Medicine, Busan, Republic of Korea; Center for Health Promotion and Cancer Prevention, Dongnam Institute of Radiological and Medical Sciences, Busan, Republic of Korea; Department of Internal Medicine, Inje University Haeundae Paik Hospital, Busan, Republic of Korea; Departments of Family Medicine, Inje University Haeundae Paik Hospital, Busan, Republic of Korea

**Keywords:** Healthcare inequality, Regional health planning, Diabetes mellitus, Hypertension

## Abstract

**Background:**

This study investigated whether the prevalence, awareness, treatment, and control of hypertension and diabetes differed by residential areas. In addition, the rate of good hypertension or diabetes control was examined separately in men and women, and in urban and rural areas.

**Methods:**

This study used Korea National Health and Nutrition Examination V (2010–2012) data, a nationwide cross-sectional survey of general South Korean population. Residential areas were categorized into urban and rural areas.

To examine differences between the residential areas in terms of prevalence, awareness, treatment, and control of hypertension and diabetes we performed a multivariate logistic regression adjusting for age, body mass index, physical activity, alcohol use, smoking, marital status, monthly income, and educational level. To investigate control of hypertension or diabetes within each residential area, we performed a subgroup analysis in both urban and rural areas.

**Results:**

The prevalence of hypertension is higher among men in urban areas than among those in rural areas (OR = 0.80; 95 % CI = 0.67–0.96, reference group = urban areas). However, the subgroups did not differ in terms of diabetes prevalence, awareness, treatment, and control. Regardless of both sex and residential area, participants in good control of their hypertension and diabetes were younger. Inequality in good control of hypertension was observed in men who lived in urban (≤Elementary school, OR 0.74, 95 % CI 0.60–0.92) and rural areas (≤Elementary school, OR 0.67, 95 % CI 0.46–0.99). Inequality in health status was found in women who resided in urban areas (≤Elementary school, OR 0.53, 95 % CI 0.37–0.75). Good control of diabetes also showed inequalities in health status for both men (≤Elementary school, OR 0.61, 95 % CI 0.40–0.94; Middle/High school, OR 0.69, 95 % CI 0.49–0.96) and women in urban areas (≤1 million won, OR 0.56, 95 % CI 0.33–0.93) (Reference group = ‘≥College’ for education and ‘>3 million’ Korean won for income).

**Conclusions:**

After correction for individual socioeconomic status, differences by residential area were not observed. However, when the participants with good disease control were divided by region, inequality was confirmed in urban residents. Therefore, differentiated health policies to resolve individual and regional health inequalities are necessary.

## Background

Multiple studies have established that social inequality in Korean society affects individual inequalities in health status [1–5]. In Health Inequalities in South Korea, 2009 published by the Korean Society for Equity in Health, people of higher educational levels had consistently lower mortality rates for both men and women in 1995, 2000, and 2005 [3]. People with low levels of socioeconomic status had more prevalent hypertension and received less treatment [6, 7]. If people had lower socioeconomic status, they had higher morbidity and mortality of cerebrovascular disease associated with hypertension [8–10]. Diabetes was also prevalent in people with low socioeconomic status in addition to hypertension [11, 12]. Diabetic patients with low socioeconomic status were hospitalized and visited emergency rooms more frequently [13].

Inequality in health status existed by region as well as individual socioeconomic status [3, 5, 14]. The prevalence of chronic diseases was higher in people who lived in rural areas than in urban areas [15]. In a study about diabetes, its prevalence was higher in people who lived in urban areas than in rural areas [16]. However, previous studies that investigated health inequalities by residential areas were focused on mortality or prevalence of chronic disease. There are few studies regarding the treatment and control of specific diseases by residential areas. This study investigated the differences by residential areas on the prevalence, awareness, treatment, and control of hypertension and diabetes in Koreans. In addition, to investigate control of hypertension or diabetes within each residential area, we performed a subgroup analysis in both urban and rural areas.

## Methods

### Data sources and study participants

This study used data from the Korea National Health and Nutrition Examination Survey (KNHANES) V (2010–2012), a nationwide survey that assessed the health and nutritional status of 25,534 Koreans. Among these, 17,292 individuals (7033 men and 9398 women) who were aged 30 years or older and completed both a health interview and health examination were identified as study participants.

The KNHANES representing the Korean general population and conducted by the Ministry of Health and Welfare and the Korea Centre for Disease Control and Prevention. It is composed of three component surveys: a health interview, a health examination, and a nutrition survey. Trained medical staff and interviewers performed the health interviews and examinations. Health behaviors such as cigarette smoking, alcohol use, and physical activity were collected via self-report. Information about medical conditions, education, income, health care utilization, activity limitation, and quality of life were identified via face-to-face interviews. Health examinations consisted of anthropometric measures such as heights, weights, blood pressures, and biochemical profiles using fasting blood serum and urine. The KNHANES used a multi-stage clustered probability design. In KNHANES V, 192 primary sampling units (PSUs) were drawn from approximately 200,000 geographically defined PSUs for the entire country. From the PSUs, 3800 households were selected. The 2010, 2011, and 2012 KNHANES response rates were 77.5, 76.1, and 75.9 % respectively. Each participant provided informed consent prior to inclusion in the study and KNHANES V was approved by the institutional review board of KCDC (2010-02CON-21-C).

### Basic questionnaire and health examination

Blood pressure was measured with a mercury sphygmomanometer (Baumanometer Desk model 0320; WA Baum Co. Inc., USA) in the sitting position after a 5 min rest period. The participants were asked to refrain from smoking or caffeine for 30 min before the measurement. The first Korrotkoff sound was used for systolic blood pressure and the fifth Korrotkoff sound was used for diastolic pressure. Three measurements were taken at 30 s intervals and we used the average of the latter two in the analysis. Body mass index (BMI) was calculated by dividing weight in kilograms by height in meters squared. Blood samples were collected after 8-h fasting to obtain laboratory results such as fasting glucose and hemoglobin A1C.

Marital status was categorized into married and others; the others category included divorced and widowed individuals. Smoking status was categorized current smoker, former smoker, and never smoker. Alcohol use was estimated from the frequency of high-risk alcohol consumption and categorized as never, less than once per month, and at least once per month. In the KNHANES V, high-risk alcohol use was defined by gender. Men who drank more than seven cups of alcohol at a single event and women who drank more than five cups of alcohol at a single event were classified as individuals with high-risk drinking. Regular physical exercise was defined as the participants who did five or more times of moderate activity or three or more times of vigorous activity per week.

Subjective health status was classified into three levels by responses to the question “How do you assess your own health status?”: poor, fair, or good. Medical accessibility was categorized in two levels by responses to the question “Could you go to the clinic (except dental clinic) when you wanted to go there during the last year?”. If the answer to this question was “yes”, we asked why the participants could not visit the clinic (economic problem or traffic inconvenience, etc.).

### Social economic status and residential areas

This study used monthly household income and education as main indicators of socioeconomic status (SES). With regard to educational attainment, the participants were asked the level at which their education was completed. This was classified into three educational categories: completion of elementary school, middle and high school, and post-secondary school . Household income was calculated based on equivalized income (total household income divided by the square root of the number of household members) and classified into three categories: ≤ 1 million won, 1–3 million won, or > 3 million won.

Residential areas were categorized into *dong*, *eup* and *myeon* in KNHANES. That is according to the Korean Local Government Act., the country consists of Metropolitan cities and provinces. Metropolitan cities are divided into districts, and provinces are divided by cities and counties. Cities and districts include multiple *dong* and counties include multiple *eup* and *myeon*. In this study, we classified *dong* as urban areas, and *eup* and *myeun* as rural areas, respectively.

### The definitions of prevalence, awareness, management, and control of hypertension and diabetes

Hypertension prevalence was defined as one of the following: SBP ≥ 140 mmHg and/or DBP ≥ 90 mmHg, receiving antihypertensive treatment, or an answer of “yes” to the question, “Have you ever been diagnosed with hypertension by a physician?” Hypertension awareness was defined an answer of “yes” to the question, “Have you ever been diagnosed with hypertension by a physician?” Hypertension treatment was defined as use of an antihypertensive drug more than 20 days per month. Hypertension control was defined SBP < 140 mmHg and DBP < 90 mmHg.

Diabetes prevalence was defined as one of the following: fasting glucose ≥ 126 mg/dL, use of oral hypoglycemic agents or insulin therapy, or an answer of “yes” to the question, “Have you ever been diagnosed with diabetes by a physician?” Diabetes awareness was defined an answer of “yes” to the question, “Have you ever been diagnosed with diabetes by a physician?” Diabetes treatment was defined an answer of “yes” to the question, “Are you now using oral hypoglycemic agents or insulin therapy?” Diabetes control was defined hemoglobin A1c < 6.5 %.

### Statistical analysis

The characteristics of study participants are presented as mean (SD) or as numbers and percentages. The prevalence of hypertension and diabetes were calculated by direct age standardization (10-year intervals) and 95 % confidence intervals (CIs) for urban and rural residents stratified by sex with the 2009 Korean residence registration population. The proportion of awareness, treatment, and control of hypertension or diabetes were calculated as the proportions of participants with hypertension or diabetes who were aware of, were taking medications for, and had well controlled hypertension or diabetes. Odds ratios (ORs) and 95 % confidence intervals (CIs) for prevalence, awareness, treatment, and control for each residential area were estimated by multivariate logistic regression analysis adjusted for age, body mass index, physical activity, alcohol use, smoking, marital status, and socioeconomic status including monthly income and educational attainment.

Multivariate logistic regression was employed to examine the odds of good control of hypertension or diabetes for each residential area. We adjusted for age, body mass index, physical activity, alcohol use, smoking, marital status, monthly income, and educational level.

All data were analyzed separately for men and women, and also for urban and rural areas. The analysis reflects the complex survey sample design, which includes stratification, clustering, and weight. All analyses were performed using SPSS ver. 18.0 (SPSS Inc., Chicago, IL, USA) and differences were considered significant at *p* < 0.05.

## Results

### Participant characteristics

The characteristics of the individuals are shown in Table 1 by residential area and sex. There were significant differences in SES with regard to residential areas in both sexes. Individuals living in rural areas had lower SES than those who lived in urban ones.

**Table 1:** Characteristics of participants by residential areas

	Male					Female		
	Total	Urban	Rural	*P-*value	Total	Urban	Rural	*P*-value
	(*n* = 7,033, *N* = 1.54^a^)	(*n* = 5,440, *N* = 1.21^a^)	(*n* = 1,593, *N* = 0.33^a^)		(*n* = 9,398, *N* = 1.61^a^)	(*n* = 7,333, *N* = 1.26^a^)	(*n* = 2,065, *N* = 0.35^a^)	
Age, years				<0.001				<0.001
30–44	41.0 (0.9)	43.7 (1.0)	31.3 (2.1)		37.5 (0.8)	40.6 (0.9)	26.1 (1.6)	
45–64	44.0 (0.8)	43.4 (0.9)	45.9 (2.1)		41.9 (0.7)	42.4 (0.8)	40.4 (1.4)	
> 64 years	15.0 (0.5)	12.8 (0.5)	22.8 (1.1)		20.6 (0.5)	17.1 (0.5)	33.4 (1.4)	
BMI, kg/m^2^	24.2 (0.05)	24.3 (0.1)	23.9 (0.1)	0.01	23.7 (0.05)	23.6 (0.06)	24.2 (0.1)	<0.001
Educational level				<0.001				<0.001
≤ Elementary school	15.2 (0.6)	11.8 (0.6)	27.4 (1.8)		31.0 (0.7)	25.2 (0.8)	52.4 (2.3)	
Middle/high school	47.5 (0.5)	46.2 (0.9)	52.0 (1.9)		44.8 (0.4)	47.0 (0.8)	36.8 (1.8)	
≥ College	37.3 (0.9)	41.9 (1.0)	20.6 (2.0)		24.2 (0.7)	27.8 (0.9)	10.8 (1.4)	
Monthly income (thousand won^b^)				<0.001				<0.001
≤ 1000	23.8 (0.8)	20.7 (0.8)	34.8 (1.9)		30.3 (0.8)	26.4 (0.8)	44.5 (1.8)	
1001–3000	59.4 (0.8)	61.7 (0.9)	50.9 (1.7)		54.8 (0.8)	57.7 (0.9)	44.2 (1.7)	
> 3000	16.9 (0.7)	17.6 (0.8)	14.3 (1.2)		14.9 (0.6)	15.9 (0.7)	11.3 (1.1)	
Marital status				0.719				<0.001
Married	83.6 (0.7)	83.4 (0.8)	84.0 (1.4)		76.4 (0.6)	77.7 (0.6)	71.7 (1.5)	
others^c^	16.4 (0.7)	16.6 (0.8)	16.0 (1.4)		23.6 (0.6)	22.3 (0.6)	28.3 (1.5)	
Smoking status				0.524				0.067
Current	46.7 (0.8)	46.5 (0.9)	47.1 (1.3)		6.1 (0.3)	6.2 (0.4)	5.8 (0.8)	
Ex-smoker	38.0 (0.7)	38.3 (0.8)	36.6 (1.6)		5.6 (0.3)	6.0 (0.3)	4.0 (0.6)	
Non-smoker	15.4 (0.5)	15.1 (0.6)	16.3 (1.1)		88.4 (0.4)	87.8 (0.5)	90.2 (1.0)	
Alcohol drinking^d^				<0.001				<0.001
Rarely or never	30.5 (0.7)	29.1 (0.8)	35.9 (1.5)		68.2 (0.6)	66.9 (0.7)	73.2 (1.5)	
< 1/month	15.4 (0.5)	16.0 (0.6)	13.2 (1.1)		15.0 (0.5)	15.2 (0.6)	14.2 (1.2)	
≥ 1/month	54.1 (0.8)	54.9 (0.8)	51.0 (1.8)		16.8 (0.5)	17.9 (0.6)	12.6 (1.0)	
Regular physical exerciser	48.6 (0.8)	48.7 (0.8)	48.5 (1.8)	0.939	42.7 (0.7)	43.2 (0.7)	41.0 (1.7)	0.261
Medical accessibility				0.026				<0.001
No	85.5 (0.6)	86.2 (0.6)	82.8 (1.5)		78.5 (0.6)	80.2 (0.6)	72.4 (1.5)	
Yes	14.5 (0.6)	13.8 (0.6)	17.2 (1.5)		21.5 (0.6)	19.8 (0.6)	27.6 (1.5)	
				0.017				<0.001
Economic reasons	13.7 (1.3)	14.9 (1.6)	10.2 (2.3)		24.6 (1.3)	25.7 (1.6)	21.5 (2.4)	
Traffic inconvenience	1.7 (0.6)	0.9 (0.5)	4.2 (1.8)		5.4 (0.6)	2.3 (0.5)	13.6 (2.0)	
Others	84.6 (1.4)	84.3 (1.7)	85.5 (3.1)		70.1 (1.4)	72.0 (1.6)	64.9 (2.8)	

Data are % (standard error [SE]) or mean (standard deviations [SD])

*Abbreviations*: *BMI* body mass index

Data are weighted to the residential population of Korea

^a^n: unweighted sample size, N: weighted sample size in millions

^b^The exchange rate is approximately 1100 Korean won for 1 US dollar

^c^Including widowed and divorced persons

^d^The frequency of high risk drinking, high risk drinking is defined as consuming more than 7 standard drinks per each occasion on average

Men living in rural areas had lower BMI with fewer unhealthy alcohol users than those who lived in urban areas. Women living in rural areas had higher BMI with fewer unhealthy alcohol users than those who lived in urban areas. Individuals who lived in rural areas had more unmet health care needs than those who lived in urban areas. The participants who could not go to hospitals because they were poor were more common in urban residents and because of traffic inconvenience were more common in rural residents (Table 1).

### Health inequalities by residential area

After adjusting for age, BMI, physical activity, alcohol, marital status, monthly income, and educational attainments, odds ratios for prevalence, awareness, treatment, and control of hypertension and diabetes classified by residential areas and sex were calculated. There were no differences between residential areas except there were fewer male hypertensive patients living in rural areas than those who lived in urban areas (Table 2).

**Table 2:** Multivariate odds ratios^a^ (95 % CI) for prevalence, awareness, treatment, and control of hypertension and diabetes according to residential areas

	Hypertension		Diabetes	
	Urban	Rural	Urban	Rural
Male				
Prevalence	1	0.80 (0.67–0.96)	1	1.05 (0.82–1.36)
Awareness	1	0.95 (0.70–1.29)	1	1.03 (0.63–1.67)
Treatment	1	1.04 (0.76–1.41)	1	1.30 (0.85–1.99)
Control	1	1.01 (0.76–1.33)	1	0.89 (0.57–1.40)
Female				
Prevalence	1	0.84 (0.70–1.02)	1	0.82 (0.64–1.04)
Awareness	1	1.00 (0.78–1.28)	1	0.76 (0.46–1.18)
Treatment	1	1.10 (0.85–1.43)	1	0.89 (0.60–1.33)
Control	1	1.03 (0.83–1.28)	1	1.04 (0.65–1.67)

Data are % (95 % confidence interval)

Data are weighted to the residential population of Korea

Urban and Rural groups are defined as participants who reside ‘*dong*’, ‘*eup* and *myeon*’ (Korean administrative section), respectively

^a^Adjusted for age, BMI, physical activity, alcohol, smoking, marital status, monthly income, and educational level

### Health inequalities of subjects with good control of hypertension and diabetes by residential areas

Regardless of both sex and residential area, more patients with good control of hypertension were in the “30–44 years” and “45–65 years” age groups than in the “older than 65 years” group. Good control of male hypertensive patients residing in both areas was less for lower educational attainment (urban, ≤Elementary school, OR 0.74, 95 % CI 0.60–0.92; rural, ≤Elementary school, OR 0.67, 95 % CI 0.46–0.99). Good control of female hypertensive patients only residing in urban areas was less for lower educational attainment (≤Elementary school, OR 0.53, 95 % CI 0.37–0.75) (Reference group = ≥College) (Table 3).

**Table 3:** Multivariate odds ratios (95 % CI)^a^ for good control of hypertension according to residential areas

	Male		Female	
	Urban	Rural	Urban	Rural
Age, years				
30-44	3.55 (2.95–4.27)	3.14 (2.11–4.69)	7.45 (5.23–10.60)	4.74 (2.47–9.07)
45-64	1.42 (1.23–1.63)	1.63 (1.26–2.13)	1.73 (1.38–2.16)	1.69 (1.17–2.42)
>64 years	1	1	1	1
Educational level				
≤Elementary school	0.74 (0.60–0.92)	0.67 (0.46–0.99)	0.53 (0.37–0.75)	0.45 (0.18–1.15)
Middle/high school	0.94 (0.81–1.10)	0.75 (0.51–1.09)	0.77 (0.57–1.03)	0.53 (0.21–1.32)
≥College	1	1	1	1
Monthly income (thousand won^b^)				
≤ 1000	0.83 (0.67–1.03)	1.08 (0.73–1.60)	0.98 (0.73–1.33	1.13 (0.65–1.99)
1001-3000	0.90 (0.76–1.07)	1.02 (0.71–1.46)	1.09 (0.83–1.42)	1.44 (0.85–2.44)
> 3000	1	1	1	1
Marital status				
Married	1	1	1	1
Others^c^	0.91 (0.78–1.07)	0.75 (0.57–0.98)	0.98 (0.79–1.20)	0.80 (0.56–1.16)
Smoking status				
Current	0.70 (0.60–0.82)	0.87 (0.68–1.11)	1.10 (0.74–1.62)	1.01 (0.60–1.70)
Ex-smoker	0.70 (0.61–0.81)	0.81 (0.62–1.07)	0.90 (0.62–1.29)	1.30 (0.71–2.39)
Non-smoker	1	1	1	1
Alcohol drinking^d^				
Non-drinker	1.74 (1.50–2.01)	1.57 (1.20–2.06)	1.31 (1.02–1.69)	1.01 (0.60–1.70)
<1/month	1.63 (1.33–2.00)	1.59 (1.10–2.32)	1.25 (0.89–1.76)	1.30 (0.71–2.39)
≥1/month	1	1	1	1
Regular physical exerciser	1.00 (0.89–1.14)	0.89 (0.70–1.11)	0.92 (0.77–1.09)	0.77 (0.57–1.02)

Data are weighted to the residential population of Korea

Urban and Rural groups are defined as participants who reside ‘dong’, ‘eup and myeon’ (Korean administrative section), respectively

^a^Adjusted for age, BMI, physical activity, alcohol, smoking, marital status, monthly income, and educational level

^b^The exchange rate is approximately 1,100 Korean won for 1 US dollar

^c^Including widowed and divorced persons

^d^The frequency of high risk drinking, high risk drinking is defined as consuming more than 5 standard drinks of women and 7 standard drinks of women per each occasion on average

In urban areas, more patients with good control of diabetes were in the “30–44 years” and “45–64 years” age groups than in the “older than 65 years” group. In rural areas, more patients with good control of diabetes were in the “30–44 years” group than in the “older than 65 years” group. Good control of male diabetes patients residing in urban areas was less for lower educational attainment (≤Elementary school, OR 0.61, 95 % CI 0.40–0.94; Middle/High school, OR 0.69, 95 % CI 0.49–0.96). Good control of female diabetes patients who resided in urban areas was less for lower income (≤1 million, OR 0.56, 95 % CI 0.33–0.93) (Reference group = ‘≥College’ for education and ‘>3 million’ Korean won for income) (Table 4).

**Table 4:** Multivariate odds ratios (95 % CI) a for good control of diabetes according to residential areas

	Male		Female	
	Urban	Rural	Urban	Rural
Age, years				
30-44	6.02 (3.75–9.66	4.77 (1.95–11.67)	6.95 (4.11–11.76)	3.77 (1.35–10.53)
45-64	1.75 (1.28–2.40)	1.58 (0.94–2.66)	1.49 (1.10–2.00)	1.19 (0.69–2.07)
>64 years	1	1	1	1
Educational level				
≤Elementary school	0.61 (0.40–0.94)	1.05 (0.49–2.23)	0.68 (0.41–1.13)	0.42 (0.11–1.59)
Middle/high school	0.69 (0.49–0.96)	0.80 (0.41–1.58)	0.80 (0.49–1.30)	0.67 (0.20–2.31)
≥College	1	1	1	1
Monthly income (thousand won^b^)				
≤ 1000	0.88 (0.57–1.37)	2.05 (0.93-4.56)	0.56 (0.33-0.93	0.78 (0.36-1.72)
1001-3000	1.08 (0.75-1.57)	1.60 (0.89-2.88)	0.66 (0.41-1.07)	0.86 (0.40-1.85)
> 3000	1	1	1	1
Marital status				
Married	1	1	1	1
others^c^	1.15 (0.76–1.75)	1.26 (0.60–2.66)	0.85 (0.64–1.12)	1.09 (0.61–1.96)
Smoking status				
Current	0.59 (0.39–0.91)	0.81 (0.41–1.59)	0.87 (0.52–1.47)	0.71 (0.29–1.73)
Ex-smoker	0.79 (0.51–1.22)	1.06 (0.55–2.03)	1.62 (0.93–2.82)	0.67 (0.21–2.15)
Non-smoker	1	1	1	1
Alcohol drinking^d^				
Non-drinker	0.84 (0.63–1.12)	1.33 (0.81–2.17)	0.67 (0.44–1.04)	0.34 (0.13–0.95)
<1/month	0.91 (0.63–1.32)	1.48 (0.70–3.14)	0.79 (0.44–1.41)	0.66 (0.21–2.10)
≥1/month	1	1	1	1
Regular physical exerciser	1.22 (0.94–1.57)	1.02 (0.69–1.50)	1.05 (0.80–1.37)	0.92 (0.57–1.49)

Data are weighted to the residential population of Korea

Urban and Rural groups are defined as participants who reside ‘dong’, ‘eup and myeon’ (Korean administrative section), respectively

^a^Adjusted for age, BMI, physical activity, alcohol, smoking, marital status, monthly income, and educational level

^b^The exchange rate is approximately 1,100 Korean won for 1 US dollar

^c^Including widowed and divorced persons

^d^The frequency of high risk drinking, high risk drinking is defined as consuming more than 5 standard drinks of women and 7 standard drinks of women per each occasion on average

## Discussion

Using KNHANES V as representative national data, the present study investigated whether residential areas influenced the prevalence, awareness, treatment, and control of hypertension and diabetes. In addition, we examined whether there were differences in health inequality between urban and rural areas with respect to good control of hypertension and diabetes. After adjusting for individual socioeconomic status, there were few differences between urban and rural areas. However, participants who lived in urban areas had consistently worse control of hypertension and diabetes with lower socioeconomic status.

Our results demonstrate that old individuals with hypertension and diabetes were in poor control of these diseases. As regards hypertension, this result was consistent with previous studies [17, 18]. Aging is related to higher blood pressure and is a major risk factor for hypertension [19]. Furthermore, the process is associated with impaired endothelium-dependent vasorelaxation, arterial remodeling, increased stiffness, and vascular inflammation, all of which are relevant to the pathophysiology of hypertension [20]. Because of age-related vascular change, older patients may have difficulty controlling their hypertension.

Previous studies have shown that longer duration of diabetes is associated with good glycemic control [21–24]. Therefore, the direct relationship between age and poor glycemic control in our study is inconsistent with other studies, in which youth was associated with poor glycemic control. [23, 25, 26]. In another study that used age rather than duration of diabetes, greater age was an independent risk factor for poor glycemic control, which is consistent with our study [27]. Therefore, the age differences in our study may have arisen from the longer duration of diabetes.

It is well known that hypertension [7, 28, 29] and diabetes [30, 31] are more common in individuals of low socioeconomic status. The impact of socioeconomic status on hypertension and diabetes might be due to socioeconomic status differences in unhealthy behaviors of smoking, physical inactivity, etc. [32, 33] or to neuroendocrine hormone changes suffered from chronic stress [34, 35]. Individuals with lower educational attainment adopted more unhealthy behaviors of smoking, unhealthy alcohol use, physical inactivity, obesity, and lack of sleep (2.97 times more in men and 2.96 times more in women) than those who graduated from the university [33].

Previous studies about urbanization level and health status reported different results. Studies conducted in Holland [36], the UK [37], and the US [38] showed that the mortality in rural areas was lower than that in urban areas, but the mortality in urban areas was lower than that in rural areas in studies conducted in China [39] and South Korea [40, 41]. There was little difference in prevalence of hypertension and diabetes compared to mortality; patients of hypertension and diabetes were more common in rural areas [41]. This study found out that male hypertensive patients who lived in rural areas were less common than those who lived in urban areas. This result was different from the previous study [41]. This difference might be caused by improving behaviors such as quitting smoking, reducing unhealthy alcohol use, and exercise in rural areas. There had been fatter men, more smokers, and unhealthy alcohol users in rural areas than those in urban areas [41]. However, in the current study, there were thinner men and fewer unhealthy alcohol users in rural areas than those in urban areas. Smoking status was not different between urban and rural areas. Actually comparing the age-standardized mortality of metabolic syndrome from 2008 to 2011, the mortality in rural areas was twice as high as that in urban areas in 2008. The mortality was still high in rural areas, but these differences had no statistical significance in 2011 [40].

Regardless of both sex and residential area, inequalities in both hypertension and diabetes control occurred. However, these differences were only significant in the following subgroups: hypertensive men in both urban and rural areas, hypertensive women, diabetic men and women in urban areas. It was well known that there were inequalities of hypertension control [42] and diabetes prevalence [16, 43] by socioeconomic status in urban areas. However, the studies about health inequalities in urban areas were mostly conducted in low-income countries [44–49]. A comparison of urban and rural areas by average indices indicates that while urban areas are known to be wealthier and healthier than rural areas due to abundant service resources, inequality within urban areas is even more severe than in rural areas [50]. In 47 countries, the stunting and mortality rates of infants residing in urban and rural areas showed more severe inequality for urban residents than for rural residents [51]. In terms of differences in socioeconomic status related to subjective health status and physical activity restriction, the poor in urban areas reported that they were unhealthy and had restrictions on their physical activities more than in rural areas [52].

The reason why inequality in hypertension and diabetes control were apparently observed more in subjects who lived in urban areas than those who lived in rural areas in this study was thought to be different effects on use of medical services. The urban poverty index was related to the number of people using medical services; however, the rural poverty index was not [53]. Unmet health care needs among rural residents were more common than urban residents in this study. However, rural residents could not use medical services because of long distances; urban residents could not use medical services because of poverty (Table 1). While differences in health care accessibility between urban and rural areas because of long distances were observed, general health conditions were not different between urban and rural residents [54]. Therefore, to improve hypertension and diabetes control and relieve inequality of hypertension and diabetes management, administrators should consider counterplans by residential areas. They can help urban poor to receive economic assistance and rural residents to improve infrastructures for geographic distances.

There are several limitations of this study. First, this was a cross-sectional study and the interpretation of the causes and results is limited. Because it included subjects with low socioeconomic status as a result of disease, there is the possibility of reverse causation bias. Second, as previously mentioned, regions classified as *eup*, *myeon*, and *dong*, which were Korean administrative districts instead of the index including population density, district financial status, and so on. However, the analyses based on administrative districts can help to understand regional characteristics and can encourage local level policies for health care services [53]. Actually, community health plans are implemented based on the administrative districs according to the law for community health in South Korea. In addition, previous studies [54, 55] have examined regional differences by using the administrative units (classified as eup, myeon, and dong). However to overcome this limitation, further studies should consider including another determinant of regional poverty, or examine regional differences among smaller administrative units. Third, population structures in rural areas were quite different from those in urban areas, especially in terms of age distribution. As mentioned in the factors associated with poor glycemic control, even though we adjusted for age, other confounding factors may be related to age; namely, duration of the diseases.

Despite these limitations, our study contributes to the understanding of regional differences in hypertension and diabetes control in South Korea. While numerous studies have analyzed health inequalities, ours examined not only the prevalence, but also the treatment and control of hypertension and diabetes. These specific factors will help clinicians and investigators to understand these diseases.

## Conclusions

The differences by residential area were not apparent after correction for individual socioeconomic status. However, when the subjects with good disease control were divided based on region, inequality was confirmed in urban residents. In addition, older individuals had poorer control of hypertension and diabetes, regardless of both sex and region. Therefore, to improve hypertension and diabetes control, as well as relieve inequalities in the management of these diseases, administrators should consider the elderly in their counterplans based on residential area. Such plans may help the poor in urban areas to receive economic assistance, as well as increase the number of clinics in rural areas, where residents must currently cover greater distances for proper healthcare.

### Abbreviations

BMI, body mass index; CI, confidence intervals; KNHANES, Korea National Health and Nutrition Examination; OR, odds ratios; PSUs, primary sampling units; SES, socioeconomic status

## References

[1] Jung-Choi K, Kim YM (2013). Socioeconomic inequalities in health status in Korea. J Korean Med Assoc.

[2] Kim YM, Kim MH (2007). Health inequalities in Korea: current conditions and implications. J Prev Med Public Health.

[3] Jung-Choi K, Kim MH, Kim YM, Sohn JI, Choi YJ. Health inequalities in South Korea, 2009. Seoul: The Korean Society for Equity in Health; 2011.

[4] Cho HJ (2013). Equity in health care: current situation in south Korea. J Korean Med Assoc.

[5] Yoon TH (2010). Regional health inequalities in Korea; the status and policy tasks. J Crit Soc Policy.

[6] Morenoff JD, House JS, Hansen BB, Williams DR, Kaplan GA, Hunte HE (2007). Understanding social disparities in hypertension prevalence, awareness, treatment, and control: the role of neighborhood context. Soc Sci Med.

[7] Cha SH, Park HS, Cho HJ (2012). Socioeconomic disparities in prevalence, treatment, and control of hypertension in middle-aged Koreans. J Epidemiol.

[8] Kaplan GA, Keil JE (1993). Socioeconomic factors and cardiovascular disease: a review of the literature. Circulation.

[9] Gulliford MC, Mahabir D, Rocke B (2004). Socioeconomic inequality in blood pressure and its determinants: cross-sectional data from Trinidad and tobogo. J Kum Hypertens.

[10] Grotto I, Huerta M, Sharabi Y (2008). Hypertension and socioeconomic status. Curr Opin Cardiol.

[11] Rabi DM, Edwards AL, Southern DA (2006). Association of socio-economic status with diabetes prevalence and utilization of diabetes care services. BMC Health Serv Res.

[12] Lee DS, Kim YJ, Han HR (2013). Sex differences in the association between socio-economic status and type 2 diabetes: data from the 2005 Korean national health and nutritional examination survey (KNHANES). Public Health.

[13] Booth GL, Hux JE (2003). Relationship between avoidable hospitalizations for diabetes mellitus and income level. Arch Intern Med.

[14] Jeong BG, Jung KY, Kim JY (2006). The relationship between regional material deprivation and the standardized mortality ratio of the community residents aged 15–64 in Korea. J Prev Med Public Health.

[15] Lee MS (2005). Health inequalities among Korean adults; socioecenomic status and residential area differences. Korean J Sociol.

[16] Lee HY, Won JC, Kang YJ (2010). Type 2 diabetes in urban and rural districts in Korea: factors associated with prevalence difference. J Korean Med Sci.

[17] Borzecki AM, Glickman ME, Kader B, Berlowitz DR (2006). The effect of age on hypertension control and management. Am J Hypertens.

[18] Cushman WC, Ford CE, Cutler JA (2002). Success and predictors of blood pressure control in diverse north American settings: the antihypertensive and lipid-lowering treatment to prevent heart attack trial (ALLHAT). J clin hypertens (Greenwich, Conn).

[19] Wang M, Monticone RE, Lakatta EG (2010). Arterial aging: a journey into subclinical arterial disease. Curr Opin Nephrol Hypertens.

[20] Harvey A, Montezano AC, Touyz RM. Vascular biology of ageing-Implications in hypertension. J Mol Cell Cardiol 2015;83:112–21.:10.1016/j.yjmcc.2015.04.011. 10.1016/j.yjmcc.2015.04.011 PMC4534766 25896391

[21] Crowley MJ, Holleman R, Klamerus ML, Bosworth HB, Edelman D, Heisler M (2014). Factors associated with persistent poorly controlled diabetes mellitus: clues to improving management in patients with resistant poor control. Chronic Illn.

[22] Khattab M, Khader YS, Al-Khawaldeh A, Ajlouni K (2010). Factors associated with poor glycemic control among patients with type 2 diabetes. J Diabetes Complications.

[23] Shan S, Gu L, Lou Q (2015). Evaluation of glycemic control in patients with type 2 diabetes mellitus in Chinese communities: a cross-sectional study. Clin Exp Med.

[24] Jeon JY, Kim DJ, Ko SH (2014). Current status of glycemic control of patients with diabetes in Korea: the fifth Korea national health and nutrition examination survey. Diabetes metab j.

[25] Benoit SR, Fleming R, Philis-Tsimikas A, Ji M (2005). Predictors of glycemic control among patients with type 2 diabetes: a longitudinal study. BMC Public Health.

[26] El-Kebbi IM, Cook CB, Ziemer DC, Miller CD, Gallina DL, Phillips LS (2003). Association of younger age with poor glycemic control and obesity in urban African Americans with type 2 diabetes. Arch Intern Med.

[27] Chen R, Ji L, Chen L (2015). Glycemic control rate of T2DM outpatients in china: a multi-center survey. Med Sci Monit.

[28] Le C, Chongsuvivatwong V, Geater A (2007). Contextual socioeconomic determinants of cardiovascular risk factors in rural south-west china: a multilevel analysis. BMC Public Health.

[29] Hoang VM, Byass P, Dao LH, Nguyen TK, Wall S (2007). Risk factors for chronic disease among rural Vietnamese adults and the association of these factors with sociodemographic variables: findings from the WHO STEPS survey in rural Vietnam, 2005. Prev Chronic Dis.

[30] Brown AF, Ettner SL, Piette J (2004). Socioeconomic position and health among persons with diabetes mellitus: a conceptual framework and review of the literature. Epidemiol Rev.

[31] Hwang J, Shon C (2014). Relationship between socioeconomic status and type 2 diabetes: results from Korea national health and nutrition examination survey (KNHANES) 2010–2012. BMJ Open.

[32] Paek KW, Chun KH, Jin KN, Lee KS (2006). Do health behaviors moderate the effect of socioeconomic status on metabolic syndrome?. Ann Epidemiol.

[33] Kim HJ, Ruger J (2010). Socioeconomic disparities in behavioral risk factors and health outcomes by gender in the republic of Korea. BMC Public Health.

[34] Brunner EJ, Hemingway H, Walker BR (2002). Adrenocortical, autonomic, and inflammatory causes of the metabolic syndrome: nested case–control study. Circulation.

[35] Williams RB (2010). How does lower education get inside the body to raise blood pressure? what can we do to prevent this?. Hypertension.

[36] van Hooijdonk C, Droomers M, Deerenberg IM, Mackenbach JP, Kunst AE (2008). Higher mortality in urban neighbourhoods in the Netherlands: who is at risk?. J Epidemiol Community Health.

[37] Senior M, Williams H, Higgs G (2000). Urban–rural mortality differentials: controlling for material deprivation. Soc Sci Med.

[38] House JS, Lepkowski JM, Williams DR (2000). Excess mortality among urban residents: how much, for whom, and why?. Am J Public Health.

[39] Zimmer Z, Kaneda T, Spess L (2007). An examination of urban versus rural mortality in china using community and individual data. J Gerontol B Psychol Sci Soc Sci.

[40] Shin J. Amenable mortality and medical expenditure according to residential areas. Health, Welfare Issue & Focus: Korea Institute for Health and Social Affairs; 2014.

[41] Yoon TH, Kim JH (2006). Health inequalities between rural and urban areas in south Korea. J Korean Acad Rural Health Nurs.

[42] Awuah RB, Anarfi JK, Agyemang C, Ogedegbe G, Aikins A (2014). Prevalence, awareness, treatment and control of hypertension in urban poor communities in Accra, Ghana. J Hypertens.

[43] Patil RR. Urbanization as a determinant of health: a socioepidemiological perspective. Soc Work Public Health 2014;29:335–41. 10.1080/19371918.2013.821360 24871771

[44] Bhojani U, Devedasan N, Mishra A, De Henauw S, Kolsteren P, Criel B (2014). Health system challenges in organizing quality diabetes care for urban poor in south India. PLoS One.

[45] Bortz M, Kano M, Ramroth H, Barcellos C, Weaver SR, Rothenberg R, Magalhães M. Disaggregating health inequalities within Rio de Janeiro, Brazil, 2002-2010, by applying an urban health inequality index. Cad Saude Publica. 2015;31(1):107–19. 10.1590/0102-311X00081214 PMC4727964 26648367

[46] Acharyya T, Kaur P, Murhekar MV (2014). Prevalence of behavioral risk factors, overweight and hypertension in the urban slums of north 24 parganas district, west Bengal, India, 2010. Indian J Public Health.

[47] Bhojani U, Mishra A, Amruthavalli S, et al. Constraints faced by urban poor in managing diabetes care: patients’ perspectives from South India. Glob Health Action 2013;6:22258.:10.3402/gha.v6i0.22258. 10.3402/gha.v6i0.22258 PMC3790910 24093885

[48] Bhojani U, Beerenahalli TS, Devadasan R, et al. No longer diseases of the wealthy: prevalence and health-seeking for self-reported chronic conditions among urban poor in Southern India. BMC Health Serv Res 2013;13:306.:10.1186/472-6963-13-306. 10.1186/1472-6963-13-306 PMC3751059 23938172

[49] Anand K, Shah B, Yadav K (2007). Are the urban poor vulnerable to non-communicable diseases? a survey of risk factors for non-communicable diseases in urban slums of Faridabad. Natl Med J India.

[50] WHO, UN Habitat. Hidden cities: Unmasking and overcoming health inequities in urban settings. 2010. http://www.who.int/kobe_centre/publications/hiddencities_media/who_un_habitat_hidden_cities_web.pdf?ua=1. (Assessed 10 Aug 2015).

[51] Van de Poel E, O'Donnell O, Van Doorslaer E (2007). Are urban children really healthier? evidence from 47 developing countries. Soc Sci Med.

[52] Yang W, Kanavos P. The less healthy urban population: income-related health inequality in China. BMC public health 2012;12:804.:10.1186/471-2458-12-804. 10.1186/1471-2458-12-804 PMC3563496 22989200

[53] Shin HS. Regional Inequality of Health Service Utilization. Korean Institute for Health and Social Affairs. 2012. https://www.kihasa.re.kr/web/publication/periodical/issue_view.do?pageIndex=18&keyField=&key=&menuId=50&tid=38&bid=21&aid=195&ano=24. (Assessed 10 Aug 2015).

[54] Kim SY, Yoon KC. An analysis of the regional differences of health inequality and the exploration of the factors causing the differences. The Korean Journal of Local Government Studies,2011:31–57.

[55] Lee JH, Paeng KY, Kim JR, Jeong BG, Park KS (2012). Self-rated health and individual level social capital across the administrative sections. Korean J Health Educ Promot.

